# Healthcare Professionals, Post-traumatic Stress Disorder, and COVID-19: A Review of the Literature

**DOI:** 10.3389/fpsyt.2021.795221

**Published:** 2022-01-21

**Authors:** Valeria Saladino, Vincenzo Auriemma, Valeria Campinoti

**Affiliations:** ^1^Department of Human, Social and Health, University of Cassino and Southern Lazio, Cassino, Italy; ^2^Department of Political and Social Studies, University of Salerno, Fisciano, Italy; ^3^Italian Center for Single Session Therapy, Rome, Italy

**Keywords:** COVID-19, healthcare professionals, PTSD, empathy, distress, hospital, infection

## Abstract

The recent COVID-19 pandemic impacted healthcare professionals psychologically. They were unprepared to handle such a powerful and unknown virus. Consequently, they had to face situations of extreme distress, developing vicarious traumatization and post-traumatic stress disorder (PTSD). The first one is associated with the “cost of caring” for others and affected persons who constantly are exposed to other sufferings. PTSD is a psychiatric disorder that could affect people who have experienced or witnessed a traumatic event. Post-traumatic stress disorder (PTSD) and correlated symptoms might impact the lives of healthcare professionals at the personal, professional, and relational levels. Furthermore, the pandemic could decrease the empathy of healthcare professionals, influencing their relationship with patients. This review aimed to describe the incidence of PTSD among HPs during the COVID-19 pandemic. We focused on the following aspects: (a) PTSD symptoms and correlated psychological issues, (b) repercussions at working and personal levels, (c) change in empathetic involvement of healthcare professionals.

## Introduction

There is a large amount of research on the psychological effect of pandemics faced by humans over time, such as the severe acute respiratory syndrome (SARS), middle east respiratory syndrome (MERS), Ebola, and COVID-19 (1, 2). These results showed negative psychological outcomes on human well-being caused by the separation from loved ones, loss of freedom, uncertainty about the state of the disease, and uneasiness. This suffering could lead to dramatic events, such as suicide, mental illness, addiction, and self-harm (3–5).

Since December 2019, we are facing a new infection, called novel coronavirus or COVID-19 (6). This infection spread out rapidly and caused mortality and contagion, producing a strong psychological impact on the population. This virus changed our everyday life, cognition, and behavior, influencing interpersonal relationships, work, and mental health. The phycological effects of COVID-19 involved all the population. However, healthcare professionals (HPs) seem to be the most affected, due to the overwork and the increased level of stress caused by the emergency. Moreover, the healthcare system was not ready to handle this emergency, leaving HPs to work with insufficient information and inadequate medical equipment. This situation has led to a gradual decimation of available human resources. HPs comprise a notable proportion of infected people (7), with a terrible impact on the healthcare system (8). In this situation, post-traumatic stress symptoms (PTSS) and post-traumatic stress disorders (PTSD) are the most common psychological issues among HPs, together with burnout syndrome (9, 10).

Burnout is a psychological breakdown derived from chronic exposure to stress and diffused among HPs (11, 12). Risk factors for clinician burnout include stressful professional experiences, increased workload, reduced quality of performance, and social isolation (13). HPs are more often severely sick. Consequently, they are obliged to avoid their families and friends, increasing the risk of post-traumatic stress disorder. This heavy emotional load may generate anxiety and depression, leading in the long term to burnout syndrome and, potentially, suicide. PTSD, instead, is a psychiatric disorder caused by a terrifying event, perceived as a trauma, which affects directly or indirectly the individual (e.g., severe accident or injury, threat to physical safety, death or threat of death, sexual assault, natural disasters, war, etc.) (14).

According to previous research on other infectious diseases, healthcare professionals experimented with anxiety, depression, and PTSD during and after the outbreak (2, 15–17). The World Health Organization in 2016 (18), published a report on Ebola outbreak survivors, stressing the risks of alcohol and tobacco addiction in those who have contracted the virus. Specifically, Maunder et al. (16) studied these risks in the HPs population during the 2003 SARS outbreak. They showed an increase in their smoking and drinking habits, higher levels of burnout and distress, and disinvestment in the relationship with patients. Furthermore, follow-up on the sample underlined that distress condition and the psychological symptoms had a long-lasting impact on the HPs' quality of life and coping strategies (Ibidem). Wu et al. (19) surveyed 549 randomly selected hospital employees in China during the 2003 SARS outbreak and confirmed these results. The authors showed a positive correlation between having been quarantined or working in high-risk wards and alcohol abuse/dependence symptoms. This association remained significant after 3 years from the outbreak. The sample seems to use alcohol as a coping strategy to decrease their PTSD symptoms. Specifically, the hyperarousal was the PTSD indicator more associated with alcohol use.

The SARS and 2014 Ebola virus pandemics were more contained than the current COVID-19 one. Therefore, it is not surprising if the same, or even worse effect, occurs among HPs (20). Indeed, the uncontrollable nature of COVID-19, its unknown treatment, its high rate of mortality, and the prolonged exposure to stress, may cause psychological issues among the medical staff (21). Moreover, the fear of infection and the consequent measure of quarantine and social distancing led to a progressive physical and emotional detachment from others (22). This condition can influence the relationship between medical staff and patients and decreases empathy. During the pandemic, HPs experimented with an emotional closeness with patients. Professionals may feel unprepared to manage their and others' feelings in a moment in which they are considered heroes. Indeed, they should save lives and be emotionally available, although probably just as worried and frightened as anyone else. This condition can provoke a high sense of psychological pressure.

Considering the relevance of the topic and recent publications regarding COVID-19 and PTSD and related symptoms in the HPs population, our review aimed to describe the incidence of PTSD among HPs during the COVID-19 pandemic, taking into account its long-term effects. Also, the paper pursued the goal to stimulate reflections on an underestimated topic, the role of empathy in the healthcare professionals-patient relationship during this pandemic.

## Materials and Methods

### Search Strategy

We selected Google Scholar, PsycINFO, PubMed, and Jstor as electronic databases to achieve the purpose of the study, due to their function to evaluate scientific articles indexed in the specific search topic and to search through a filter by keywords compared with the publication dates, as well as the possibility of finding the full text of the articles. These features made the research usable and rigorous. We searched the databases according to the main topics we were interested in: (1) PTSD symptoms and correlated psychological issues in HPs, (2) empathetic involvement of healthcare professionals, (3) long-term effect of COVID-19 in HPs.

The following keywords were selected for the first topic: “COVID-19” OR “COVID-19 outbreak” OR “COVID-19 pandemic” OR “COVID-19 virus” OR “COVID-19 infection” OR “COVID-19 disease” AND “stress” OR “anxiety” OR “depression” OR “PTSD” OR “PTSS” OR “vicarious traumatization” OR “secondary traumatic stress” OR “trauma” OR “burnout” in healthcare professionals” OR “healthcare personnel” OR “HPs” OR “medical staff” OR “HCPs” OR “HW.”

The second topic was explored using the same keywords for “COVID-19” AND the following keywords: “relationship doctor/patient” OR “healthcare professional-patient relationship” OR “sociology of health” OR “agape” OR “empathy” OR “empathetic relationships in hospital” OR “empathetic relationships in hospital wards.”

The third topic was explored using the same keywords for “COVID-19” AND the following keywords: “long-term effect” OR “long-term consequences” in healthcare professionals” OR “healthcare personnel” OR “HPs” OR “medical staff” OR “HCPs.”

### Inclusion and Exclusion Criteria

The review focused on PTSD and correlated symptoms in HPs, the long-term effect of PTSD and psychological distress of COVID-19 in HPs, and empathy in HPs-patients relationship. We included articles and reviews in the English language published between 2020 and 2021. We did not comprehend geographical constraints in the searching procedure.

Articles published in a language other than English and articles out of topic were excluded.

### Study Selection

**Figure 1:**
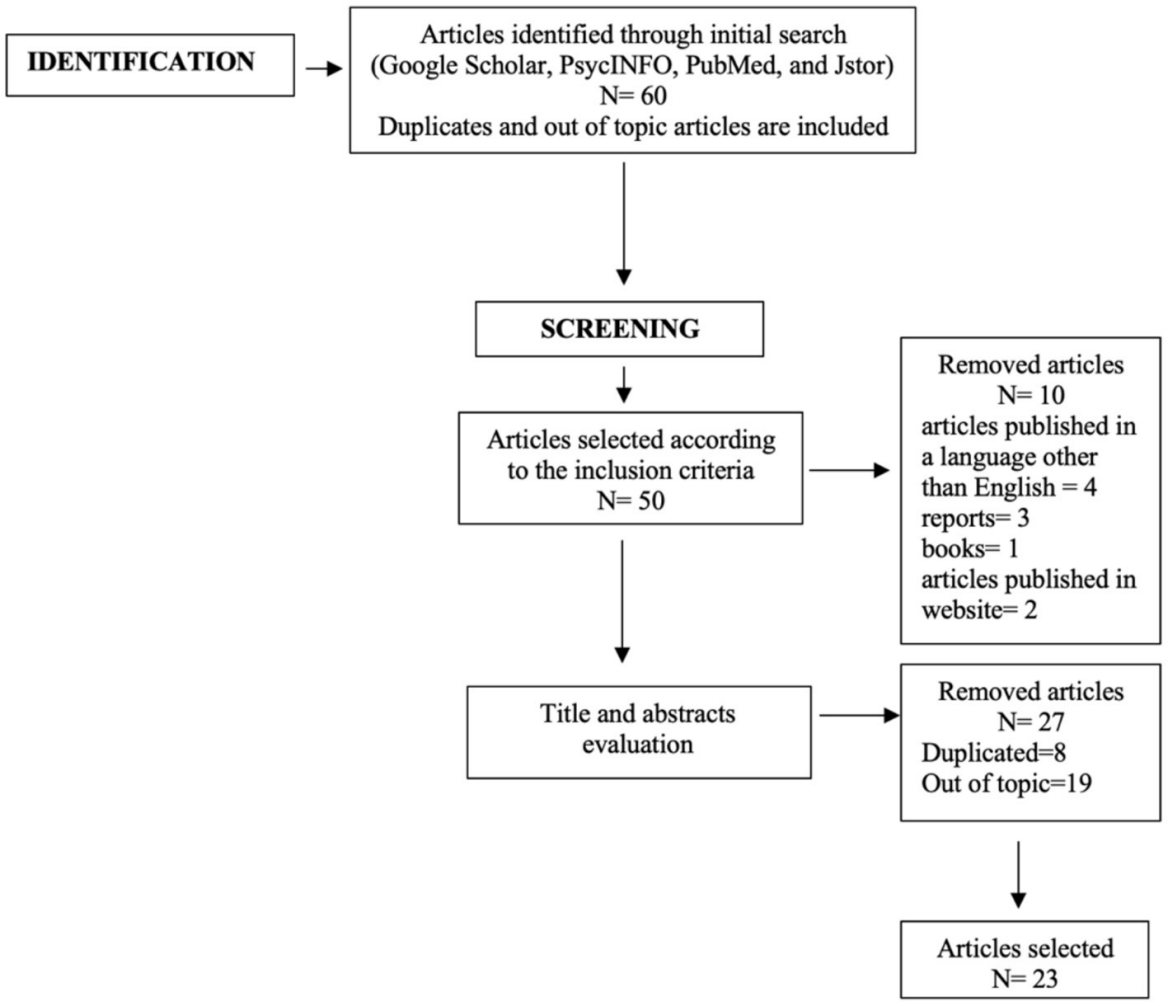
Flowchart describing the search strategy.

The study selection procedure was structured in two steps, as shown in the flowchart (Figure 1). We included 23 articles of the 60 identified (Table 1).

**Table 1:** Authors, year of publication, country, and targets of articles selected for the review.

**References**	**Country**	**Targets**
Bassi et al. (23)	Italy	Physicians, nurses and midwives, professionals in technical and rehabilitation areas, and healthcare assistants
Blekas et al. (24)	Greece	Different medical specialties and nursing staff
Braquehais et al. (25)	China, Italy, Jordan, Singapore, India	HPs working in the first line of care, HPs in quarantine, physicians, medical staff hospital, doctor, health personnel, nurses
Cao et al. (26)	China	Doctors, nurses, and technicians
Chen et al. (27)	China	Medical staff
Chirico et al. (9)	Italy	Medical staff
Dai et al. (28)	China	Doctors and nurses
Di Tella et al. (29)	Singapore, China	Medical staff
Di Tella et al. (10)	Italy	Doctors, nurses, midwives, psychologists, laboratory technicians, and administrative workers
Duncan (30)	United Kingdom	Nurses
Ghebreyesus (31)	United States	Medical staff
Gray and Back (32)	United States	Doctor-patient relationships
Guo et al. (21)	China, India, Singapore, Iran, Pakistan, Jordan, Bahrain, Hong Kong, Israel, Nepal, Oman, Saudi Arabia, South Korea), Italy, Turkey, Switzerland, Serbia, Ireland, Argentina, Brazil, Chile and Mexico, Brazil, North America.	Nurses, physicians, and doctors
Huang et al. (33)	China	Clinical first-line medical staff
Jalili et al. (7)	Iran	Physicians, residents, interns, and nurses
Kang et al. (34)	China	Medical staff
Kisely et al. (35)	China, Taiwan, Canada, Hong Kong, Singapore South Korea, Saudi Arabia, Greece, Mexico, Japan, the Netherlands, Germany, Liberia	Medical staff
Lai et al. (36)	China	Nurses, physicians, and frontline health care workers
Li et al. (37)	China	Licensed registered nurses who worked in hospitals and general public.
Pearman et al. (38)	United States	Doctors, nurses, psychologists, laboratory technicians
Rossi et al. (39)	Italy	Nurses, physicians, and general practitioner
Smith et al. (40)	United Kingdom	Healthcare practitioners
Wilson et al. (41)	India	Medical staff

In the first step, we identified articles according to the inclusion and exclusion criteria. In the second step, the titles and abstracts were screened, and the out of topic articles or duplicates records were removed.

## Results

### Post-traumatic Stress Disorder and Related Symptoms in Healthcare Professionals During COVID-19 Pandemic

During the recent COVID-19 pandemic, HPs have been in direct contact with patients, taking care of several potentially infectious people, and experimenting with physical and psychological pressure (36). Although HPs are trained to have a high tolerance toward difficult situations, they can be at risk of vicarious traumatization or secondary traumatic stress (37, 42). Indeed, health emergencies are associated with higher risk for HPs in developing post-traumatic stress disorder (PTSD) and correlated symptoms (43, 44) such as physical and emotional exhaustion, depersonalization, and dissociation (43, 45).

PTSD is included in the Diagnostic and Statistical Manual of Mental Disorders (DSM-5) in the category of Trauma- and Stressor-Related Disorders. PTSD develops following a traumatic event, in which a person has experienced or witnessed one or more events that threatened their own or others' physical integrity, experiencing intense fear, feelings of helplessness, and anxiety (14). According to the Manual, PTSD is characterized by the following symptoms and must last for more than one month. The traumatic event is experienced through intrusive flashbacks, memories, nightmares which cause emotional distress and physical arousal. The person tends to avoid all trauma-related stimuli, such as thoughts, emotions, place, and other external reminders, progressive worsening of negative feelings about oneself, sense of blame and guilt for the trauma, apathy, self-isolation, aggression, insomnia, hypervigilance (Ibidem).

As reported by some studies (46, 47) the measures adopted and the fear of the infection, impacted people's lifestyles. These feelings generate high levels of psychological distress and may lead to traumatic experiences.

Braquehais et al. (25) summarized the main risks factors for HPs in developing PTSD and correlated symptoms during the COVID-19 pandemic:

- *COVID-19 exposure:* HPs who work in the COVID-19 ward have high responsibilities and are more likely to be infected. They live a constant fear of contagion and fear to transmit the infection to their families. In most cases, HPs accompany dying patients alone due to preventive measures, living vicarious traumatic experiences.
- *Epidemiology:* The rate of virus incidence influences the level of anxiety and depression among HPs. Furthermore, the trend of the growth case numbers is proportional to the intensity of work in COVID-19 wards. When HP's overwork, they feel less confident in their capacity to manage stress and support patients.
- *Public health policies:* The lack of data transparency from the government on the number of infections and the ambiguity of prevention and treatment plans in hospitals influence the emotional distress and the feeling of loneliness and abandonment from the Institutions.
- *Material resources:* The shortage of resources, such as personal protection equipment, products to sanitize environments, place in which to rest after hours of work, increase the fear of contagion among HPs, as well as decrease the perceived support of the healthcare system.
- *Human resources:* The health emergency and the consequent lack of human resources led to greater intensity in working hours and increasing daily commitment. HPs suffer from emotional and physical stress that has become chronic, leading to psychological exhaustion.
- *Personal factors:* HPs who have children are more likely to experiment with the fear of infecting them. Also, people who have psychological issues and feelings of loneliness have less resilience in managing the stress and ineffective coping strategies. They could resort to self-medication, such as substance use, incrementing the risk of developing an addiction.

China was the first place in which the pandemic developed. Dai et al. (28) collected data from a sample of 1.704 Chinese HPs. The researchers found a high level of psychological distress, anxiety, somatization, obsessive-compulsive, phobic anxiety, and psychoticism among them than the control group (25).

In the same line, Cao et al. (26) involved 5.062 HPs and found that 29.8% of them reported stress, 13.5% depression, and 24.1% anxiety. Chen et al. (27) explored the personal perception of security and fear on 13 physicians in a Chinese hospital during the COVID-19 outbreak, finding that the main worry for them was to infect their family members, the inability to manage patients' panic, and the shortage of protective equipment. Lai et al. (36) confirmed these results in a survey on 1.257 HPs who worked in COVID-19 wards, among 34 hospitals in China. They found a high level of anxiety (44.6%), depression (50%), distress (71.5%), and insomnia (34%). These symptoms can lead to obsessive thoughts related to fear of infection and a progressive decrease in interpersonal relationships (34, 41). In another study conducted in China on 230 HPs, the incidence of PTSD was estimated at 27.39% (33).

An evaluation of mental health outcomes among HPs in Italy during the COVID-19 pandemic showed similar results (39). These data confirmed the high level of stress-related psychological issues, particularly among frontline HPs. Bassi et al. (23) conducted a survey on HPs belonging into three categories, depending on the work setting: “Inpatient frontline” with patients in acute care, emergency, and with infectious diseases, “Inpatient second line” with patients in medical and surgical domains, and “Outpatient and services second line” which include labs, and family doctors. The survey showed that HPs who work in the frontline are more likely to develop PTSS and PTSD. On the same line, Di Tella et al. (29) compared HPs working in COVID-19 wards and other units, demonstrating that the former showed higher depressive and PTSS. Moreover, the authors find that prolonged exposure to traumatic events, such as the death of patients and colleagues, might lead to PTSD.

Similarly, Pearman et al. (38) conducted an online survey among 35 states in the US, to evaluate the psychological impact of COVID-19 on HPs. The comparison between 90 HPs and the control group showed higher levels of depression, tiredness, anxiety, health worrying, lower coping strategies in managing stress. These symptoms reduce the quality of life and resiliency, developing chronic diseases, like burnout and PTSD. According to this research, medical staff presents higher acute PTSD than non-medical ones (35).

An online survey conducted in Greece on 270 HPs (24) showed a high score of PTSD correlated with other symptomatology, such as insomnia, depression, physical tension, and negative feelings on COVID-19. HPs with PTSD are more worried about the evolution of the virus. They think that it is a terminal disease and not predictable. These feelings are associated with the lack of experience with pandemics and the scarcity of resources. This perception of lack of control by HPs is predictive of PTSS, increasing burnout, and their self-perception of inadequate competencies.

Another factor that contributes to developing PTSD is the sense of isolation of HPs who did the quarantine after they have been in contact with an infected person, as well as HP's survivors from the infection (22). This category of HPs can feel isolated and stigmatize themselves due to the related fear of infecting their beloved ones. The consequences of COVID-19 on the health care system led clinicians and scientists to call it the “9/11 of health care systems.” HPs are asked to comply with a new health organization, to relationships with patients, to deal with suffering and illness, unexpectedly. For instance, they often need to conserve or reuse personal protective equipment due to the shortages in resources, accepting the high risk of being infected as part of their work. HPs feel need for support but at the same time they are aware to be a resource for their patients. This conflict can lead to uneasiness in medical staff. In the most exposed places, HPs must choose how to ration the resources they have available to make sure they are enough for all patients, and this causes a moral injury and a sense of injustice and deep suffering. Moreover, the absence of physical contact, imposed by social distancing, reduces the sense of closeness, resilience, optimism, and flexibility (30).

### Healthcare Professional-Patient Relationship: The Role of Empathy

Empathy is the ability to understand the emotional process of others (48) and includes an instinctual, anthropological-cultural, and historical component. HPs might decrease their level of empathy to protect themselves from suffering. Indeed, as research on mirror neurons highlighted, witnessing suffering can evoke the same discomfort in the observer (49). Thus, HPs may not bear the pressure caused by long periods of experiencing people's pain. This “forced detachment” and the progressive exclusion of empathy might damage the relationship with patients (50). The most favorable situation might be finding a balance between total detachment and excessive involvement to preserve the psychological health of HPs and patients and their relationship.

Sociology emphasizes the importance of empathy. According to Ardigò (51), empathy helps for understanding the experience of the disease, and in taking care of patients: “There cannot be good medical care if there is not an agreement between what the doctor evaluates and what the patient does or does not do” (p. 193). Ardigò (52) defines the construct of empathy as the ability to understand the emotional processes of others, or “the capacity of individual consciousness which is one of the genetic poles of social life” (p. 4).

The recent COVID-19 pandemic has radically changed the way HPs relate to patients. During an emergency, HPs can react in two opposite ways. They could project their feelings into the patients' conditions, increasing the risk of secondary traumatization (37, 42), or they might focus more on fighting the virus than on human relations.

Gray and Back (32) studied the evolution of empathic communication in the HPs-patient relationship using comics. In their study, the authors emphasize, with cartoons, how the doctor performs routine actions, almost without asking whether that action is advantageous or not. For instance, they showed the difficulties of the doctors in explaining to patients the infection by COVID-19. Empathic communication aims to strict compliance with World Health Organization directives (Ibidem).

The pandemic changed the patient-professional relationship. On the one hand, people feel considered only in the role of “patient” and there is no time and resources to pay attention to their concerns. On the other hand, HPs are afraid of not being able to manage their patients' emotions, in a state of suffering as high as that caused by COVID-19.

The evolution in communication between HPs and patients during the COVID-19 pandemic should investigate how empathy is changing to promote a healthy relationship between the medical staff and patients, decreasing distress.

### The Long-Term Consequences of COVID-19 on the Mental Health of Healthcare Professionals

Since the first period of the pandemic, the research studied the psychological consequences of the emergency on HPs, who found themselves facing an unexpected situation (31).

HPs experienced extreme working conditions, they have had no time for rest or leisure, and many have also given up family life for fear of infecting their loved ones (36). COVID-19 pandemic undermines the belief that human beings are invincible and instead puts them back in a condition of vulnerability. Disease on the global scale creates a climate of fear, insecurity, panic, anxiety, frustration, aggression, and post-traumatic symptoms (53). These emotional and psychological states are amplified among the medical staff responsible for healing and saving lives. During the pandemic, the Institutions impose new behavioral norms, such as social distancing, to reduce the spread of the infection. Instead, HPs had to move in the opposite direction, working in high-risk situations with poor protective equipment or training.

Similarly, during the SARS epidemic in 2003–2004, 18 to 57% of HPs experienced severe emotional problems and psychiatric symptoms during and after the pandemic (54). In 2015 MERS, long-term dysphoria, and stress emerged among HPs (55). These results revealed that post-traumatic stress disorder in HPs persists even after a period of absence from work. Tam et al. (2) also showed that HPs' mental health implications could be chronic, affecting many areas of their personal life. According to several studies (56–58), an event that occurs in a limited period—however severe—is less traumatic than chronic and prolonged stress over time without an end. The persistence of the invisible virus over time, with no indication on when the pandemic could diminish or end, represents a systematic trauma and requires a constant effort to process the situation (59). Furthermore, according to the American Psychiatric Association (14), people with PTSD are more likely (80%) to develop other disorders than people without PTSD. Overall, anxiety, depression, and substance abuse are the most common disorders that occur as comorbidities of PTSD.

In addition, the absence of boundaries between private and working life and the adrenaline-fueled condition allows the HPs to “dissociate” from the traumatic experience, like in wartime (53). The status of “superhero” attributed by the population and the media to HPs provoke additional pressure. Indeed, superheroes are invincible and cannot fail or be sick.

After the emergency, traumatic experiences remain “open wounds.” According to the phases of stress, people affected by PTSS and PTSD need support after the post-traumatic period, during which individuals deal with their related trauma emotions. Considering the explicit risk of burnout and post-traumatic symptoms among HPs, and the repercussions on their mental health in the long term, structured psychological interventions are needed. These interventions should aim to prevent and treat PTSS and PTSD and promote the personal empowerment of HPs, as suggested by the Istituto Superiore di Sanità (60).

## Discussion

During the COVID-19 pandemic, health systems collided with a crucial challenge in organizing human and technical resources. The research carried out in this period provided a picture of the psychological impact of COVID-19 on HPs, during the crisis and in the long term (31). Our study highlighted the high prevalence of PTSS and PTSD, especially among healthcare professionals who work in COVID-19 wards (36). They have responsibilities and are more likely to be infected, living in the constant fear of contagion. The systematic exposure to emotional distress, anxiety, and sense of isolation represents a risk factor for psychological issues that can worsen in the future.

These considerations are common within countries. Indeed, regardless of contexts, stress seems to be the most observed psychological outcome among HPs. Data from studies conducted in China on healthcare professionals showed the following psychological issues: distress, somatization, obsessive-compulsive symptoms, phobic anxiety, depression, insomnia, worrying about infecting family members, inability to manage the panic of patients infected by the virus, and lack of perceived support by healthcare system (25–28, 36). The same considerations are valid for Italy, the United States, and Greece (24, 38, 39). Moreover, these studies found also that HPs have lower coping strategies and a consequent worse quality of life, developing more often chronic diseases, like PTSD (35).

Managing stress and psychosocial well-being during this period is as important as taking care of physical health and using effective coping strategies. A break during the work shift or between shifts, eating healthy food, exercising whenever possible, and keeping in touch with family and friends. Ineffective coping strategies, such as substance use and self-isolation, should be categorically excluded. They can worsen mental and physical well-being by increasing stress. The scenario in which many operators are involved is undoubtedly unique, especially for those HPs who have never been involved in emergency events of this magnitude. It can be helpful to use strategies that have proved effective in managing stress. The World Health Organization (61, 62) recommended the following guidelines to preserve HPs from chronic stress during emergency:

- Regularly monitor the psychological well-being of the staff;
- Ensure good quality of communication and update accurate information;
- Evaluate the working times and the rest or break times that the staff needs;
- Give practitioners a space to express concerns and ask questions;
- Encourage mutual support among colleagues;
- Facilitate access to mental health services and Psychological First Aid, within the work context and outside.

Another aspect to consider is the evolution in empathic communication in healthcare professional-patient relationships. The recent COVID-19 pandemic also changed this relationship (27). Due to their emotional and physical exhaustion, HPs reduced psychological closeness and empathic communication with patients as defense mechanisms. They try to focus on fighting the virus and invest their resources in working, focusing less on the relationship (40). The conflict between being psychologically available and being an operating machine provokes a process of “depersonalization” in HPs and patients, who cannot communicate feelings to each other. In this situation, empathic communication decreases within the medical facilities. The relation between HPs and patients during the COVID-19 pandemic should be further investigated to improve psychological well-being.

### Future Directions

According to our results, future directions should be based on promoting well-being in HPs, for instance, providing them psychological support, group meeting, and briefing to discuss stressful events and situations within the workplace to manage and monitor their personal and work condition. Furthermore, another aspect to implement is the coping skill in operating daily difficulties and distress. This direction can also be improved through constant monitoring on the medical staff, for example using interviews or questionnaires periodically to observe the quality of life in the workplace and to prevent symptoms of burnout, PTSD, anxiety, depression, and other negative outcomes.

Regarding the doctor-patient relationship, it would be useful to provide indications on the importance of empathy in the relationship with patients and to give HPs the tools necessary to positively implement the relationship with patients and mutual communication.

### Limitations

This review presents some limitations. Gender differences, types of HPs (physicians, nurses, etc.), and cultural and geographical constraints were not considered in exploring PTSD. Also, we did not consider the differences between “waves” or “states” of the pandemic, nor the impact of the pandemic in HPs according to the evolution of the virus during months. Future research should consider these aspects to analyze PTSD and the long-term effects on health professionals to structure targeted interventions.

## Conclusion

It is certainly not possible to propose exhaustive conclusions in the field of HPs' mental health during the COVID-19 pandemic. However, the literature analyzed suggests some reflections and questions foster a debate in the future. A first reflection is related to the development of strategies to address psychological challenges related to the pandemic, focusing on HPs and communities, also considering their long-term consequences. The second consists in realizing a careful and targeted approach for the public mental health service. The last reflection regards the study of the impact of the virus on the healthcare professional-patient relationship, focusing on the role of empathy. HPs work hardly in an emergency, and they need support.

## Author Contributions

VS and VA conceptualized the contribution. VS, VA, and VC wrote the manuscript. VS provided the critical revision processes as PI. All authors approved the submission of the manuscript.

## Conflict of Interest

The authors declare that the research was conducted in the absence of any commercial or financial relationships that could be construed as a potential conflict of interest.

## Publisher's Note

All claims expressed in this article are solely those of the authors and do not necessarily represent those of their affiliated organizations, or those of the publisher, the editors and the reviewers. Any product that may be evaluated in this article, or claim that may be made by its manufacturer, is not guaranteed or endorsed by the publisher.
